# Chemical Compositions and Insecticidal Activities of *Alpinia kwangsiensis* Essential Oil against *Lasioderma serricorne*

**DOI:** 10.3390/molecules201219818

**Published:** 2015-12-08

**Authors:** Yan Wu, Wen-Juan Zhang, Dong-Ye Huang, Ying Wang, Jian-Yu Wei, Zhi-Hua Li, Jian-Sheng Sun, Jia-Feng Bai, Zhao-Fu Tian, Ping-Juan Wang, Shu-Shan Du

**Affiliations:** 1 Technical Center of China Tobacco Guangxi Industrial Co., Ltd., Nanning 530001, Guangxi, China; abelabel@126.com (Y.W.); huangdongye8@163.com (D.-Y.H.); jtx_wjy@163.com (J.-Y.W.); jtxlizhihua@hotmail.com (Z.-H.L.); nnsjs@sina.com (J.-S.S.); baijiafeng10@163.com (J.-F.B.); 18301243767@163.com (Z.-F.T.); 15177471944@163.com (P.-J.W.); 2 Beijing Key Laboratory of Traditional Chinese Medicine Protection and Utilization, Beijing Normal University, No. 19 Xinjiekouwai Street, Beijing 100875, China; zwj0729@mail.bnu.edu.cn (W.-J.Z.); yingw150@163.com (Y.W.)

**Keywords:** *A. kwangsiensis*, *Lasioderma serricorne*, essential oil, contact toxicity, fumigant toxicity

## Abstract

The essential oil obtained by hydrodistillation from *Alpinia kwangsiensis* rhizomes was investigated by GC-MS. A total of 31 components representing 92.45% of the oil were identified and the main compounds in the oil were found to be camphor (17.59%), eucalyptol (15.16%), β-pinene (11.15%) and α-pinene (10.50%). These four compounds were subsequently isolated and the essential oil and four isolated compounds exhibited potent insecticidal activity against *Lasioderma serricorne* adults. During the assay, it was shown that the essential oil exhibited both potential contact (LD_50_ = of 24.59 μg/adult) and fumigant (LC_50_ = of 9.91 mg/L air) toxicity against *Lasioderma serricorne*. The study revealed that the insecticidal activity of the essential oil can be attributed to the synergistic effects of its diverse major components, which indicates that oil of *Alpinia kwangsiensis* and its isolated compounds have potential to be developed into natural insecticides to control insects in stored grains and traditional Chinese medicinal materials.

## 1. Introduction

The cigarette beetle, *Lasioderma serricorne* (Fabricius, Coleoptera: Anobiidae) is widely distributed around the world, especially in tropical and subtropical areas [1]. As one of the major insects in stored tobacco products, cereal grains and processed foods, *Lasioderma serricorne* causes significant losses of grains, foods or traditional Chinese medicinal materials stored in warehouses [2]. Its development and survival are closely affected by the type of food, temperature and humidity, thus, its life cycle can be quite different [1]. Control of *Lasioderma serricorne* populations around the world is primarily dependent upon continued applications of phosphine [3]. However, its repeated use for decades has led to serious problems, especially in insecticide resistance, disruption of biological control by natural enemies, environmental and human health concerns, rising cost of production and lethal effects on non-target organisms [4,5]. There is therefore an urgent need to find an alternative strategy to control this pest. Due to their abundance and natural biodegradable features, plants have played a significant role in the development of insecticides [6]. In recent years, essential oils have received a great deal of attention as pest control agents. They are typically characterized by low toxicity to human and animals, high volatility, and toxicity to stored grain insect pests [7]. In a previous report [8], essential oils were reported as applicable to the protection of stored products.

*Alpinia*, a member of the ginger family (Zingiberaceae), comprises more than 250 species found in Southeast Asia, extending from Japan in the north to Australia in the south and into the Western Pacific [9]. *Alpinia kwangsiensis* T. L. Wu et Senjen is one of the 50 species of *Alpinia* found in China. The rhizomes of *A. kwangsiensis* are used in Chinese traditional medicine for the treatment of abdominal pain, stomach cold vomiting and traumatic injury [10,11]. The chemical constituents of rhizomes of this medicinal herb have been studied [12]. During our mass screening program for new agrochemicals from the wild plants, essential oil of *A. kwangsiensis* rhizomes was found to possess strong insecticidal activity against the cigarette beetle. According to recent literature, it was shown that there are no reports on the insecticidal activity of the essential oil from *A. kwangsiensis* rhizomes. Hence, we decided to use the essential oil obtained from *A. kwangsiensis* rhizome parts and its main chemical constituents to investigate its insecticidal activities against *L. serricorne*.

## 2. Results and Discussion

### 2.1. Chemical Compounds of the Essential Oil

The essential oil was obtained by hydrodistillation from the dried rhizomes of *A. kwangsiensis* with a yield of 0.16% (*v*/*w*) and a density of 0.86 g/mL. The chemical constituents of *A. kwangsiensis* essential oil are shown in Table 1. The main constituents of *A. kwangsiensis* essential oil were camphor (17.59%), eucalyptol (15.16%), β-pinene (11.15%) and α-pinene (10.50%) followed by α-terpineol (7.28%), camphene (6.85%) and limonene (5.22%). A total of 31 components were identified in the essential oil of *A. kwangsiensis*, accounting for 92.45% of the total oil (Table 1).

**Table 1:** Chemical components of the essential oil from *A. kwangsiensis*.

Peak No.	Components	RI ^a^	%RA ^b^	Identification Methods ^c^
**1**	Tricyclene	919	0.17 ± 0.07	MS, RI
**2**	3-Thujene	925	0.06 ± 0.02	MS, RI
**3**	Benzaldehyde	928	0.11 ± 0.09	MS, RI
**4**	α-Pinene	932	10.50 ± 0.06	MS, RI, Co
**5**	Camphene	943	6.85 ± 0.04	MS, RI
**6**	β-Pinene	974	11.15 ± 0.03	MS, RI
**7**	α-Phellandrene	999	0.18 ± 0.12	MS, RI
**8**	Benzyl alcohol	1008	0.09 ± 0.04	MS, RI
**9**	β-Terpinene	1013	2.86 ± 0.02	MS, RI
**10**	Eucalyptol	1017	15.16 ± 0.03	MS, RI
**11**	Limonene	1040	5.22 ± 0.02	MS, RI
**12**	γ-Terpinene	1060	0.20 ± 0.05	MS, RI
**13**	Terpinolene	1083	0.26 ± 0.03	MS, RI, Co
**14**	Linalool	1090	1.16 ± 0.02	MS, RI
**15**	Perillene	1094	1.05 ± 0.04	MS, RI
**16**	Campholenic aldehyde	1112	0.71 ± 0.05	MS, RI, Co
**17**	Camphor	1120	17.59 ± 0.06	MS, RI
**18**	Borneol	1159	3.16 ±0.05	MS, RI
**19**	Terpinen-4-ol	1162	1.60 ± 0.12	MS, RI
**20**	Myrtenal	1166	0.38 ± 0.10	MS, RI
**21**	α-Terpineol	1174	7.28 ± 0.02	MS, RI
**22**	Myrtenol	1180	3.25 ± 0.03	MS, RI
**23**	Bornyl acetate	1289	0.58 ± 0.06	MS, RI
**24**	Methyl geranate	1307	0.11 ± 0.10	MS, RI
**25**	Neryl alcohol	1347	0.45 ± 0.03	MS, RI
**26**	α-Caryophyllene	1454	0.39 ± 0.09	MS, RI
**27**	Germacrene D	1480	0.19 ± 0.06	MS, RI
**28**	Valencen	1484	0.32 ± 0.02	MS, RI
**29**	Myristicin	1489	0.84 ± 0.06	MS, RI
**30**	Spathulenol	1578	0.16 ± 0.07	MS, RI
**31**	τ-Cadinol	1617	0.42 ± 0.04	MS, RI
	Monoterpenoids		82.86	
	Sesquiterpenoids		0.9	
	Aromatic compounds		7.83	
	Ester compounds		0.69	
	others		0.17	
	Total		92.45 ± 0.06	

^a^ Retention index (RI) relative to the homologous series of *n*-alkanes on the HP-5 MS capillary column. ^b^ Relative area (peak area relative to the total peak area). ^c^ MS = mass spectrum, Co = co-injection with standard compound.

However, the principal components of essential oil from *A. kwangsiensis* rhizomes analyzed in this work differed from those in previous report, for example, in the previous study, cinnamic acid methyl ester was the main isolated and identified component, making up 94.54% of the essential oil from *A. kwangsiensis* [12], a fact possibly due to differences in the place of origin and plant parts used.

### 2.2. Insecticidial Activity

The essential oil of *A. kwangsiensis* rhizome parts showed contact toxicity against *L. serricorne* adults with an LD_50_ value of 24.59 μg/adult (Table 2). Compared with the positive control, pyrethrins, the essential oil showed much less contact toxicity on *L. serricorne* adults. However, compared with other essential oils in the literature, for example, the essential oil of *Litsea cubeba* (LD_50_ = 27.33 μg/adult), the essential oil of *A. kwangsiensis* possessed stronger contact toxicity against *L. serricorne*, [13].

The essential oil of *A. kwangsiensis* rhizomes also possessed fumigant activity against *L. serricorne* with an LC_50_ value of 9.91 mg/L air (Table 2). However, the currently used grain fumigant, phosphine was reported to have fumigant activity against *L. serricorne* adults with LC_50_ value of 0.75 mg/L air [14]. The fumigant activity of the essential oil against the *L. serricorne* was thus 13 magnitudes lower than that of the commercial fumigant phosphine. Compared with other essential oils in previous studies, the essential oil of *A. kwangsiensis* rhizomes exhibited roughly the same level fumigant toxicity against the cigarette beetles, e.g. essential oils of *Pistacia lentiscus* (LC_50_ = 8.44 mg/L air) and *Elsholtzia stauntonii* (LC_50_ = 10.99 mg/L air) [6,15], and exhibited stronger fumigant toxicity against *L. serricorne* than the essential oil of *Agastache foeniculum* (Lamiaceae) (LC_50_ = 21.57 mg/L air) [16]. The crude essential oil of *L. muscari* aerial parts showed pronounced contact toxicity against *T. castaneum*, *L. serricorne* and *L. bostrychophila*, with LD_50_ values of 13.36, 11.28 μg/adult and 21.37 μg/cm^2^, respectively (Table 2).

Among the four main compounds, camphor and eucalyptol account for 17.59% and 15.16% of the essential oil and possessed stronger contact (LD_50_ = 11.30 and 15.58 μg/adult) and fumigant toxicity (LC_50_ = 2.91 and 5.18 mg/L air) against *L. serricorne* than that of the essential oil, β-pinene and α-pinene, respectively. What’s more, β-pinene and α-pinene exhibited weaker contact (LD_50_ = 65.87 and 77.28 μg/adult) and fumigant toxicity (LC_50_ = 35.69 and 37.57 mg/L air) against *L. serricorne* than that of the essential oil (Table 2). Thus, the contact and fumigant toxicity of the essential oil might be attributable to a synergistic effect of the sum of the pure components activity.

**Table 2:** Fumigant and contact toxicity of essential oil of *A. kwangsiensis* aerial parts and its main components against *Lasioderma serricorne* adults.

Treatments	Fumigant Toxicity			Contact Toxicity		
	LC_50_^a^ (μg/mL Air)	95% FL ^c^	Chi Square (χ^2^)	LD_50_^b^ (μg/adult)	95% FL ^c^	Chi Square (χ^2^)
*A. kwangsiensis*	9.91	8.95–11.09	20.00	24.59	21.42–28.85	19.06
Camphor *	2.91	2.57–3.26	13.11	11.30	7.78–14.07	16.13
Eucalyptol *	5.18	4.63–5.70	16.79	15.58	12.88–18.02	15.18
β-Pinene *	35.69	32.50–39.11	15.79	65.87	58.72–75.68	19.55
α-Pinene **	37.57	34.31–41.19	14.90	77.28	69.02–87.30	14.71
Phosphine	0.75	-	-	-	-	-
Pyrethrum	-	-	-	0.24	0.16–0.35	17.36

*Data from Zhang *et al.* [17]; **data from Yang *et al.* [13]. ^a^ 50% of lethal concentration, ^b^ 50% of lethal dose, ^c^ fiducial limits.

The results of this work suggest that among the four main essential oil components, camphor and eucalyptol showed stronger contact and fumigant toxicity against *L. serricorne*. In previous reports, the four components have been demonstrated to possess insecticidal activities against several stored product insects such as *Sitophilus zeamais, Tribolium castaneum, Leptinotarsa decemlineata*, and broadbean weevil [18,19,20,21]. The high volatility of these toxic compounds likely delivered fumigant toxicity by vapor action via the respiratory system, but further work is needed to confirm their extract mode of action.

## 3. Experimental Section

### 3.1. Plant Material

The fresh rhizomes (2.0 kg) of *A. kwangsiensis* were harvested from Xishuangbanna (21°08′–22°36′ N latitude and 99°56′–101°50′ E longitude), Yunnan Province, China in August 2013. The plant was identified, and a voucher specimen (BNU-dushushan-2013-08-12-25) was deposited at the Herbarium (BNU) of the College of Resources Science and Technology, Beijing Normal University.

### 3.2. Insects

Cultures of the cigarette beetle, *L. serricorne*, were maintained in the laboratory without exposure to any insecticide before use. They were reared on a sterilized diet (wheat flour/yeast, 10:1, *w*/*w*) at 29–30 °C, 70%–80% r.h. in the dark. The unsexed adult beetles used in all the experiments were about 1–2 weeks old. All containers housing insects used in experiments were made escape proof with a coating of polytetrafluoroethylene (Fluon (ICI America Inc., Bridgewater, NJ, USA)).

### 3.3. Isolation of the Essential Oil and Purification of the Four Constituents

The sample was air-dried and ground to powder using a grinding mill. The powders were subjected to hydrodistillation for 6 h using a modified Clevenger-type apparatus and extracted with *n*-hexane. Anhydrous sodium sulphate was used to remove water after extraction. The essential oil was stored in airtight containers in a refrigerator at 4 °C for subsequent experiments.

The crude essential oil (9 mL) was chromatographed on a silica gel column (45 mm i.d., 500 mm length, 160 to 200 mesh, Qingdao Marine Chemical Plant, Qingdao, Shandong Province, China) by gradient elution with mixtures of two solvents: petroleum ether and ethyl acetate (the proportion of the two solvents was changed from 100:0–0:100). Each 150 mL of eluate was collected as a fraction and concentrated at 35 °C. According to thin layer chromatography (TLC), fractions with similar profiles were combined to yield 35 fractions. Of these, fractions 3, 9, 13 and 25 were further separated by repeated silica gel column chromatography and PTLC to afford four pure compounds for determining their structure. The isolated compounds were identified based on their nuclear magnetic resonance spectra. ^1^H- and ^13^C-NMR spectra were recorded on an AMX500 instrument (Bruker-Biospin, Billerica, MA, USA) using CDCl_3_ as the solvent with TMS as internal standard. The spectral data of camphor (1.36 g, 96%) matched the data given in [22]; the spectral data of eucalyptol (0.97 g, 97%) matched that in [23]; the spectral data were also identical to the published data of β-pinene (0.56 g, 96%) [24], and α-pinene (0.33 g, 95%) [24], respectively.

### 3.4. GC-MS and GC-FID Analysis

The obtained essential oil was packed in lightless amber vials. A sample of the oil was diluted in *n*-hexane and subjected to analysis by gas chromatography coupled to a flame ionization detector (GC-FID) and gas chromatography coupled to spectrometry (GC-MS) at Beijing Normal University (Beijing, China).

GC-MS analysis was performed on a Trace DSQ instrument (Thermo Finnigan, Lutz, FL, USA) equipped with a flame ionization detector and a HP-5MS (30 m × 0.25 mm × 0.25 μm) capillary column. The column temperature was programmed at 50 °C for 2 min, then increased at 2 °C/min to the temperature of 150 °C and held for 2 min, and then increased at 10 °C/min until the final temperature of 250 °C was reached, where it was held for 5 min. The injector temperature was maintained at 250 °C and the volume injected was 0.1 mL of 1% solution (diluted in *n*-hexane). The carrier gas was helium at flow rate of 1.0 mL/min. Spectra were scanned from 50 to 550 *m/z*. Most constituents were identified by comparison of their retention indices with those reported in the literature. The retention indices were determined in relation to a homologous series of *n*-alkanes (C_5_–C_36_) under the same operating conditions. Further identification was made by comparison of their mass spectra with those stored in NIST 05 and Wiley 275 libraries or with mass spectra from reference [25]. Relative percentages of the individual components of the essential oil were obtained by averaging the GC peak area% reports.

### 3.5. Contact Toxicity

The contact toxicity of the crude essential oil and isolated compounds against *L. serricorne* adults was measured as described by Liu and Ho [26]. Range-finding studies (96.75–0.22 μg/adult, 1.5 times dilution) were run to determine the appropriate test concentrations. A serial dilution of the essential oil/compounds (five concentrations) was prepared in *n*-hexane. Aliquots of 0.5 μL of the dilutions were applied topically to the dorsal thorax of the insects. Controls were determined using *n*-hexane. Ten insects were used for each concentration and control, and the experiment was replicated five times. Both treated and control insects were then transferred to glass vials with culture media and kept in incubators. Mortality was recorded after 24 h and the LD_50_ values were calculated using Probit analysis [27] (SPSS V20.0, IBM Inc., Armonk, NY, USA). The positive control, pyrethrins (pyrethrin I and II, 37%) were purchased from Dr. Ehrenstorfer (Augsburg, Germany).

### 3.6. Fumigant Toxicity

The fumigant activity of the crude essential oil and isolated compounds against *L. serricorne* adults was tested as described by Liu and Ho [26]. Range-finding studies were run to determine the appropriate testing concentrations. A serial dilution of the essential oil/compounds (five concentrations) was prepared in *n*-hexane. A Whatman filter paper disc (diameter 2.0 cm) was impregnated with 20 μL dilution and then placed on the underside of the screw cap of a glass vial (diameter 2.5 cm, height 5.5 cm, volume 25 mL). The solvent was allowed to evaporate for 10 s before the cap was placed tightly on the glass vial, each of which contained 10 insects inside to form a sealed chamber. Fluon was used inside the glass vial to prevent insects from contacting the treated filter paper. Preliminary experiments demonstrated that 10 s was sufficient for the evaporation of solvents. *n*-Hexane was used as a control. Five replicates were carried out for all treatments and controls, and they were incubated under the same conditions as rearing. Mortality was determined after 24 h of treatment, and the LC_50_ values were calculated using Probit analysis [27] (IBM SPSS V20.0, IBM Inc., Armonk, NY, USA).

## 4. Conclusions

This study demonstrates that the essential oil obtained from *A. kwangsiensis* rhizomes and its main components (camphor and eucalyptol) exhibit significant insecticidal activity on *L. serricorne*. These findings also suggest that the bioactivities of the essential oil may be attributed to its bioactive compounds. Considering that the currently used fumigants are synthetic insecticides, the essential oil from *A. kwangsiensis* rhizomes, camphor and eucalyptol are quite promising leads, and they also show potential for development as possible natural fumigants for the control of stored product insect pests. However, for the practical application of the essential oil and compounds as novel fumigants, further studies on the safety of the essential oil and compounds to humans and on formulation development are needed.
